# Database Bias in the Detection of Interdomain Horizontal Gene Transfer Events in Pezizomycotina

**DOI:** 10.3390/biology13070469

**Published:** 2024-06-26

**Authors:** Kevin Aguirre-Carvajal, Cristian R. Munteanu, Vinicio Armijos-Jaramillo

**Affiliations:** 1 Department of Computer Science and Information Technologies, Faculty of Computer Science, University of A Coruña, Campus Elviña s/n, 15071 Coruña, Spain; kevin.aguirre@udla.edu.ec (K.A.-C.); c.munteanu@udc.es (C.R.M.); 2 Bio-Cheminformatics Research Group, Universidad de Las Américas, Quito 170513, Ecuador; 3 Carrera de Ingeniería en Biotecnología, Facultad de Ingeniería y Ciencias Aplicadas, Universidad de Las Américas, Quito 170513, Ecuador

**Keywords:** interdomain HGT, Pezizomycotina, eukaryotes, database unbalance

## Abstract

**Simple Summary:**

Bacteria increase their genetic diversity by acquiring genetic material from a variety of organisms across different species, a process known as horizontal gene transfer. Recently, there has been an increasing emphasis in the scientific literature on the importance of HGT for the evolution of eukaryotes such as fungi, animals, and plants. However, this observation presents a paradox since eukaryotes have developed mechanisms to prevent HGT. Our work proposes an alternative scenario to explain the abundance of reported cases of HGT in eukaryotes. We suggest that the misidentification of HGT candidates in eukaryotes is due to the lack of similar sequences (genes or proteins) in public databases. To support this hypothesis, we designed specific databases to identify potential HGT candidates within a particular group of fungi. Our results demonstrate that fewer HGT candidates are detected when more similar sequences exist for these candidates in databases. This finding holds significant relevance as public databases continue to grow; consequently, newly available information may refute several previously identified instances of HGT. Our experiments lead us to conclude that database imbalances need consideration before asserting new occurrences of HGT in eukaryotes.

**Abstract:**

Horizontal gene transfer (HGT) is a widely acknowledged phenomenon in prokaryotes for generating genetic diversity. However, the impact of this process in eukaryotes, particularly interdomain HGT, is a topic of debate. Although there have been observed biases in interdomain HGT detection, little exploration has been conducted on the effects of imbalanced databases. In our study, we conducted experiments to assess how different databases affect the detection of interdomain HGT using proteomes from the Pezizomycotina fungal subphylum as our focus group. Our objective was to simulate the database imbalance commonly found in public biological databases, where bacterial and eukaryotic sequences are unevenly represented, and demonstrate that an increase in uploaded eukaryotic sequences leads to a decrease in predicted HGTs. For our experiments, four databases with varying proportions of eukaryotic sequences but consistent proportions of bacterial sequences were utilized. We observed a significant reduction in detected interdomain HGT candidates as the proportion of eukaryotes increased within the database. Our data suggest that the imbalance in databases bias the interdomain HGT detection and highlights challenges associated with confirming the presence of interdomain HGT among Pezizomycotina fungi and potentially other groups within Eukarya.

## 1. Introduction

In recent years, an ongoing debate has emerged regarding the significance of interdomain horizontal gene transfer (HGT) in the evolutionary dynamics of eukaryotic genomes. Numerous studies have documented isolated instances of HGT affecting species across various major eukaryotic lineages. In particular, the literature is replete with reports of HGT within the subphylum Pezizomycotina, which belongs to one of the largest groups of Ascomycota fungi [1,2,3,4]. However, dissenting voices have cast doubt on this widespread lateral transfer of genetic material in eukaryotes [5,6,7]. These critical perspectives point to barriers that may limit HGT events in eukaryotes, such as nuclear membranes, specific intron processing mechanisms, gene-silencing processes for unpaired DNA, and distinct germ lines [8,9,10,11,12,13].

Additionally, the approach to identifying interdomain HGT has also posed challenges. For instance, there have been questions raised about relying solely on BLAST for HGT detection [6] or recovering homologs based purely on similarity before reconstructing phylogenetics [7]. Likewise, anomalous phylogenies have been identified as potential cases of HGT, leading to a growing number of reports in this field. This is significant because variable mutation rates, differential gene loss, erroneous phylogenies, and incomplete lineage sorting can potentially be misconstrued as signals of HGT [14]. To overcome these pitfalls in HGT detection, new approximations have been developed over the last few years. For instance, Ku et al. [15] suggest grouping all possible gene families in prokaryotes and eukaryotes and then examining the presence of a prokaryotic family in one or a few eukaryotic lineages as evidence of interdomain HGT. A similar approach is taken by Cote-L’Heureux et al. [1], who identify interdomain HGT events by focusing on genes where the proportion of potential recipient eukaryotes exceeds what would be expected, based on the distribution of all analyzed gene families. However, these approaches may overlook HGT instances if potential homologs to the candidate genes are excluded during the clustering steps.

After conducting horizontal gene transfer searches over several years within Pezizomycotina, we identified a potential additional bias in the detection of interdomain HGTs related to imbalanced databases. Specifically, large databases like the NCBI’s NR database contain more information from bacteria than eukaryotes. Consequently, the HGT candidates previously detected and reported are now undetectable with current database information due to the availability of more eukaryotic sequences. In light of these observations, we investigated how changing the proportion of eukaryotic species in the database affects our ability to recover interdomain HGT candidates in Pezizomycotina.

## 2. Methods

An experiment was constructed to explore how the quantity of eukaryotic sequences in a database impacts the identification of horizontal gene transfer occurrences in Pezizomycotina fungi originating from bacterial sources. The composition of the database was used as an independent variable, consisting of four variants: 100%, 75%, 50%, and 25% of eukaryotic proteins with an unchanged proportion of bacterial sequences. The number of interdomain HGT events detected served as the dependent variable.

To establish the initial protein databases, we utilized the NCBI datasets tool v16.14.0 (https://www.ncbi.nlm.nih.gov/datasets/ accessed on 22 May 2024) to acquire bacterial and eukaryotic reference proteins in compressed form on 8 April 2024. Subsequently, the eukaryotic protein dataset was subjected to random sampling using seqtk v1.4-r122 (https://github.com/lh3/seqtk) (accessed on 8 April 2024) to model the gradual accumulation of eukaryotic sequence data over time. The sampled sub-datasets representing 75%, 50%, and 25% of the original eukaryotic information were combined with bacterial protein data, resulting in four distinct databases for further analysis. Each database was then used as input to build a Diamond v2.1.9 protein database by utilizing taxonomic information derived from the NCBI’s taxdump files: names.dmp, nodes.dmp, and prot.accession2taxid downloaded on the aforementioned date by employing the makedb command. Table 1 displays the contents of the protein databases.

To identify the species that contains the highest number of HGT candidates, we obtained 1137 proteomes of Pezizomycotina using the NCBI datasets tool v16.14.0 in dehydrated mode (downloaded on 12 April 2024). These proteomes were then employed as queries for BLASTp searches with Diamond v2.1.9 [16] in a database containing bacteria + 0.25eukaryote sequences, configured to retrieve up to 500 target sequences per query. The goal of utilizing a database with a minimal proportion of eukaryotic sequences was to maximize the detection of HGT candidates.

Subsequently, Darkhorse v2.0 [17] was applied with default parameters to analyze all Pezizomycotina proteomes using the results from Diamond v2.1.9 BLASTp searches. This initial assessment identified the Pezizomycotina proteomes with the highest frequency of interkingdom HGT occurrences. Subsequently, an analysis was conducted to identify bacterial contamination in Pezizomycotina genomes by examining suspicious contigs containing only bacterial genes using BLASTn. Proteomes showing potential bacterial contamination were eliminated from further consideration. The proteomes with over 30 HGT candidates underwent BLASTp searches in the remaining databases; however, those with insufficient HGT candidates (<30) were excluded from subsequent analyses. The output files from the BLASTp searches were then scrutinized using Darkhorse v2.0 to identify HGT candidates for each database analyzed and revealed instances of horizontal gene transfer exclusively from bacterial sources.

The data’s normal distribution and equality of variance were verified using Shapiro–Wilk and Levene’s tests. Both parametric and non-parametric tests were employed to assess the impact of databases on HGT recovery, followed by a post hoc Dunn’s test to examine differences between factors.

## 3. Results 

In this research, we propose that the disparity in database composition (with more prokaryotic than eukaryotic sequences) leads to an overestimation of interdomain HGT cases identified. To examine this assumption, we generated several databases with varying proportions of eukaryotic sequences to identify potential HGT instances.

**Figure 1:**
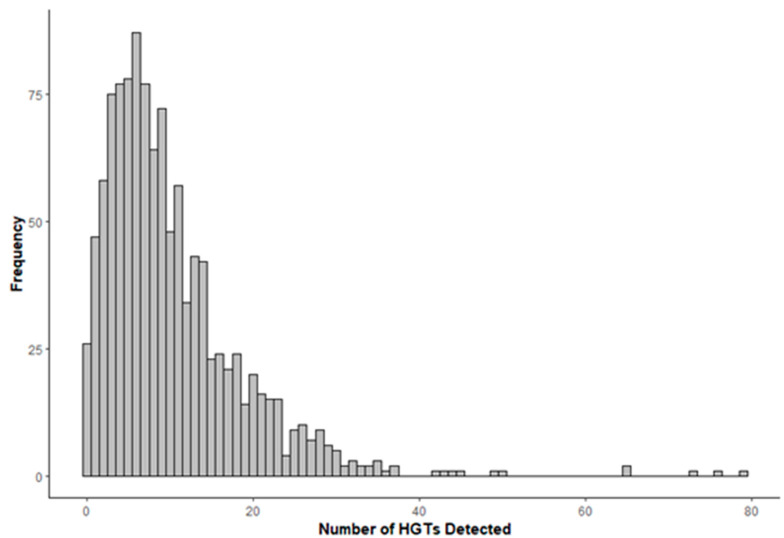
Frequency of HGT candidates detected in Pezizomycotina proteomes. The histogram exhibits a right-skewed distribution, indicating that the majority of the species have a low proportion of HGT-detectable candidates.

In the preliminary analysis for potential horizontal gene transfer cases, we analyzed 1137 proteomes from the Pezizomycotina subphylum using a database containing a low proportion of eukaryotic sequences, resulting in 15,242 identified HGT candidates (see Table S1). Following a validation step, we identified 14 proteomes with an unusually high proportion of HGT candidates. Upon examining their respective genomes, we identified contigs containing bacterial genetic material but no traces of fungal information. Suspecting potential contamination in the sequencing project, we omitted this data from further analysis. After excluding these proteomes, we found 11,227 remaining HGT candidates. Our analysis indicated that the average number of observed HGTs per proteome is approximately 10 ± 8, with approximately 98% of the examined organisms having fewer than 30 HGT candidates (Figure 1).

**Figure 2:**
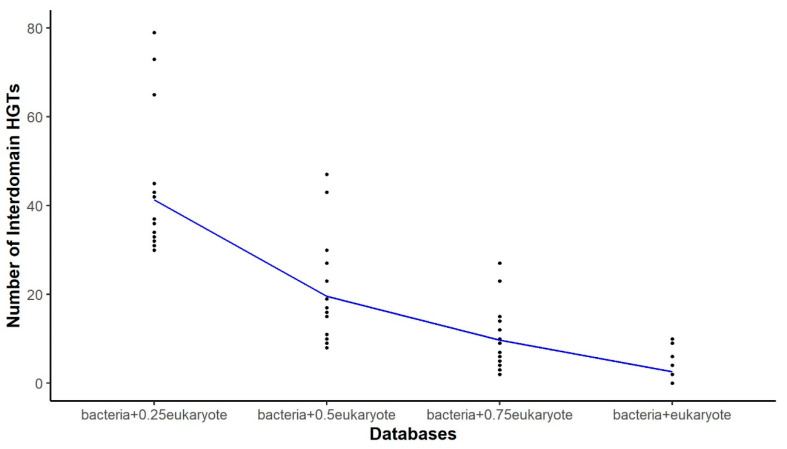
Scatterplot of interkingdom HGT events detected in different databases. The plot above illustrates the number of HGT candidates detected by Darkhorse when using databases with varying proportions of eukaryotic sequences. A blue line connects the median data collected for each database.

Based on the data obtained, we chose to conduct additional analyses using proteomes that retrieve 30 or more candidates. The impact of the database on detecting HGT candidates is illustrated in Figure 2 and Table S2.

The data indicates a clear pattern—as the proportion of eukaryotic proteins in the database increases, the number of detected HGT events decreases. For example, assembly GCA_021432635.1 shows 79 HGT events with the bacteria + 0.25eukaryote database, which decreases to 30, 14, and finally 0 with the bacteria + 0.5eukaryote, bacteria + 0.75eukaryote, and bacteria + eukaryote databases, respectively. This pattern was consistently observed across all explored proteomes. To confirm this trend, a Kruskal–Wallis test was conducted which resulted in a highly significant *p*-value of 3.15 × 10^−14^. This supports the hypothesis that the presence of eukaryotic homologs in databases affects the detection of interdomain HGT candidates and implies that including a higher proportion of eukaryotic proteins in databases leads to a notable reduction in detected HGT events.

Following the significant outcome of the Kruskal–Wallis test, a post hoc Dunn’s test was performed to determine specific pairs of database configurations that exhibited notable variations. The results revealed substantial differences in the database with a lower proportion of eukaryotic sequences (bacteria + 0.25eukaryote) compared to the other three databases. Notably, there were no significant differences observed between bacteria + 0.5eukaryote and bacteria + 0.75eukaryote or between bacteria + 0.75eukaryote and bacteria + eukaryote pairs (Table S3). These findings emphasize that there is a pronounced decrease in detected HGT candidates when comparing databases with different proportions of eukaryotic proteins. This supports earlier observations indicating that the composition of the protein reference database significantly impacts HGT detection, where higher proportions of eukaryotic proteins lead to fewer lateral gene transfer events being detected in Pezizomycotina.

## 4. Discussion

In recent years, there has been a lot of research on interdomain HGT, suggesting that this is a common phenomenon in the evolution of eukaryotes. However, it might be less common than currently depicted in the literature [5]. Assessing the impact of HGT in eukaryotes is challenging due to gaps in genome information and the incomplete collection of available eukaryotic genomes. Moreover, existing methods for detecting HGT have limitations. Although previous studies have identified biases in HGT detection [5,6,7], little attention has been paid to the effect of database composition. Salzberg et al. [18] highlighted how database composition can lead to an overestimation of potential HGT candidates in early human genome drafts. The consequence of this phenomenon is simple to predict—when a database contains fewer homologs for an HGT candidate, it becomes challenging to identify their vertical transmission history, leading to a false impression of lateral transfer.

In eukaryotes, the effect of the database in the interdomain HGT detection must be significant, produced by the unbalance between prokaryotic and eukaryotic sequences in the big public databases that are usually used as starting points to conduct searches into laterally transferred material. For instance, in the NCBI’s NR database as of 20 May 2024, only 18.51% of the sequences are from eukaryotes compared to 47.14% from bacteria. Our experimental design aimed to validate this hypothesis and found that the database significantly influences the identification of potential interdomain HGT candidates, particularly within Pezizomycotina. This effect may also hold importance for other groups of organisms and different types of HGTs such as intradomain transfers.

We acknowledge that the findings produced by Darkhorse in our experiments may not accurately represent the most credible HGT events. This software relies on BLAST searches and comparing taxonomical distances of hits to the query. This approach may not be stringent enough to determine the phylogenetic relationship between candidates and their homologs. Nonetheless, we utilized this tool to obtain a general approximation of detectable HGT candidates in complete proteomes. Furthermore, we employed the HGTector method [19] to compare Darkhorse’s results and found similar outcomes. While we recognize that rigorous methodology is essential for identifying plausible HGT candidates, our focus was primarily on investigating how databases influence HGT detection rather than providing formal descriptions of such candidates.

Considering the imbalance in databases, methods for detecting HGTs based on searching for homologs (e.g., [20,21,22]) may lead to misidentifying candidates, as observed in this study. Even approaches that balance data and create gene families before identifying candidates [1,15] could still fail to detect all available homologs or, in the worst-case scenario, sequences showing vertical inheritance patterns in certain HGT candidates may remain unavailable. Considering this reasoning, the sequences presented as potential interdomain HGT cases should be updated from time to time to verify the accuracy of the findings.

Pezizomycotina and other eukaryotic groups have been found to exhibit a high frequency of HGT events. For instance, the presence of genes from diverse kingdoms has been observed in plants [23,24,25], arthropods [26,27,28,29], and plant-parasitic nematodes [30,31,32]. However, caution must be exercised when reporting cases of HGT in eukaryotic lineages due to potential biases in database representation. Failure to do so may lead to the erroneous identification of HGT candidates instead of genuine lateral transfers. While this work focuses exclusively on data collected for Pezizomycotina, its conclusions are relevant for future studies investigating interkingdom or interdomain HGT across major eukaryotic groups.

## 5. Conclusions

Our findings strongly indicate that the imbalance in databases skews the number of interdomain HGT candidates in Pezizomycotina and likely in other eukaryotic groups. The claim that interdomain HGT in eukaryotes is common, especially in the Pezizomycotina subphylum, should be made with significant reservations. The database imbalance is inevitable, making it difficult to avoid bias in HGT detection. In light of this research, critical perspectives on HGT in eukaryotes should be taken seriously [6,7], and new approaches need to be considered to evaluate the significance of lateral genetic transfers in the evolution of eukaryotic genomes.

## Figures and Tables

**Table 1:** Composition of databases used to perform the experiments.

Database ^a^	Number of Bacterial Sequences	Number of Eukaryotic Sequences
bacteria + eukaryote	72,492,617 (42.73%)	97,191,297 (57.27%)
bacteria + 0.75 eukaryote	72,492,617 (49.87%)	72,893,472 (50.13%)
bacteria + 0.50 eukaryote	72,492,617 (59.87%)	48,595,648 (40.13%)
bacteria + 0.25eukaryote	72,492,617 (74.92%)	24,297,824 (25.08%)

^a^ 0.75%, 0.50%, and 0.25% correspond to the proportion of eukaryotic sequences in relation to the total number of eukaryotic sequences found in the bacteria + eukaryote database, which is considered as 100%.

## Data Availability

All the information is provided in the Supplementary Materials.

## Supplementary Materials

The following supporting information can be downloaded at: https://www.mdpi.com/article/10.3390/biology13070469/s1, Table S1. Number of interdomain HGT events detected in Pezizomycotina proteomes; Table S2. Number of interdomain HGT candidates detected across different databases in Pezizomycotina species; Table S3. Dunn’s test pairwise comparisons of the number of interdomain HGTs detected using different protein databases.
